# Ethnopharmacological Survey on Treatment of Hypertension by Traditional Healers in Bukavu City, DR Congo

**DOI:** 10.1155/2021/6684855

**Published:** 2021-07-09

**Authors:** Félicien Mushagalusa Kasali, Christian Ahadi Irenge, Pacifique Murhula Hamuli, Patient Birindwa Mulashe, Delphin Murhula Katabana, Jean De Dieu Mangambu Mokoso, Pius Tshimankinda Mpiana, Justin Ntokamunda Kadima

**Affiliations:** ^1^Department of Pharmacy, Faculty of Pharmaceutical Sciences and Public Health, Official University of Bukavu, Bukavu, Democratic Republic of the Congo; ^2^Pharm-Biotechnology and Traditional Medicine Center of Excellence, Department of Pharmacy, Mbarara University of Science and Technology, Mbarara, Uganda; ^3^Department of Internal Medicine, Faculty of Medicine, Official University of Bukavu, Bukavu, Democratic Republic of the Congo; ^4^Department of Biology, Faculty of Science and Applied Sciences, Official University of Bukavu, Bukavu, Democratic Republic of the Congo; ^5^Department of Chemistry, Faculty of Science, University of Kinshasa, Kinshasa, Democratic Republic of the Congo; ^6^School of Medicine, College of Medicine and Health Sciences, University of Rwanda, Kigali, Rwanda

## Abstract

**Background:**

Ethnopharmacological studies are relevant for sustaining and improving knowledge of traditional medicine within the framework of complementary/alternative therapeutic practices based solely on experience and observation across generations. Hypertension is a common cardiovascular disorder affecting more than 50% of older people in Africa (PLoS One. 2019; 14 (4): e0214934; published online on April 5, 2019, doi: 10.1371/journal.pone.0214934).

**Methods:**

We conducted a cross-sectional survey from October 2014 to August 2015 with 18 renowned traditional healers from the city of Bukavu to capture botanical plant species and remedies used by herbalists to manage hypertension in the Democratic Republic of Congo.

**Results:**

Respondents cited 41 plant species belonging to 25 botanical families. The ten most common plants are *Allium sativum*, *Galinsoga ciliata*, *Moringa oleifera*, *Bidens pilosa*, *Persea americana*, *Piper capense*, *Catharanthus roseus*, *Rauvolfia vomitoria*, *Sida rhombifolia*, and *Vernonia amygdalina*. The parts used are primary leaves (48.8%) formulated as oral decoctions (65.9%).

**Conclusion:**

The literature review validated the use of 73.2% of the plants listed. Plants of high local use-value not supported by other studies deserve in-depth chemical and pharmacological studies.

## 1. Introduction

Hypertension (HT) is a permanent rise in blood pressure with systole > 140 mmHg and diastole > 90 mmHg, resulting from arterial disturbance of the vascular tree [1, 2]. Numerous molecular signaling entities are part of the pathogenesis of HT, presuming a homeostatic imbalance [1, 2]. Several pathological events can increase the relationship between the thickness of the vascular wall and the dimensions of the arterial lumen. These can be increased arterial responsiveness (sensitivity and potency) due to deregulation of endothelial nitric oxide synthase (eNOS) and prooxidant enzymes, increased basal and activated calcium levels due to transmembrane permeability of calcium (VSMC), hyperplasia, and hypertrophy (vascular remodeling). HT represents the most growing component of the burden of cardiovascular disease (CVD). Globally, around 40% of adults aged 25 and over suffer from HT, which represents more than a billion people [3], mainly among the black population in urban and rural communities in sub-Saharan Africa [4–6].

For centuries, herbal remedies have played a central role in managing HT and other cardiovascular diseases. The discovery of highly active synthetic drugs has somewhat clouded herbal medicine, especially in wealthy Western countries. In poor rural communities in Africa, herbal remedies remain an essential part of traditional medicine, which is part of complementary/alternative medicine (CAM) [7–9]. The popularity of CAMs in African populations relates to more straightforward accessibility at a lower cost than Western therapies [10]. Patients receive social treatment from friends, relatives, and traditional healers with previous good experiences. The positive results are due to the placebo or the actual efficacy [10]. It is also interesting to note that there is currently a revival of CAM in developed countries.

The rationale for using herbal remedies comes as no surprise, given that they contain thousands of bioactive components that have known therapeutic applications [11]. A diverse range of plant and herbal extracts and their metabolites can modulate signaling cascades implicated in cardiovascular physiology. Various plants have provided a starting point for synthesizing more than 50% of the pharmaceutical drugs currently in use [12]. Cases of ephedrine (*Ephedra sinica*), aspirin (*Salix alba*), lovastatin (*Monascus purpureus*), and reserpine (*Rauvolfia serpentine*) are illustrative [12]. Reserpine depletes adrenergic neurotransmitters and remains an effective treatment for HT [13] in association with other medicines.

This study aimed to explore how local healers in Bukavu city in the eastern Democratic Republic of Congo (DRC) manage HT with plants. A recent survey in Bukavu [14] showed that 9.7% (189/1948) of hospitalized patients, 57% men and 43% women aged 23–88 years, presented with a hypertensive crisis. The majority had severe HT with BP > 180/110, including 24.4% urgency and 76% emergency. Target organ damage was stroke (32%), heart failure (24.6%), chronic kidney disease (19.7%), and whether or not associated with diabetes (39.8%); comorbidities were respiratory distress, urinary tract infections, sepsis, malaria, gastritis, and cancer. Some patients recognized having combined traditional and modern medication. Ethnopharmacological surveys are considered essential for the acquisition and protection of ancestral medical heritage. In addition, evidence-based scientific studies are worth taking to validate the efficacy claimed by traditional healers and to determine bioactive chemicals.

## 2. Methods

### 2.1. Study Design and Area

**Figure 1:**
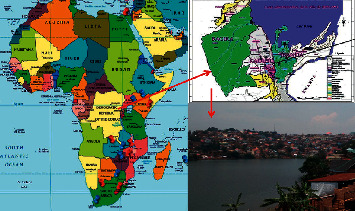
Map of Bukavu city in the Democratic Republic of Congo.

The study was a cross-sectional survey conducted in the city of Bukavu with 18 renowned traditional healers from October 2014 to August 2015 to capture botanical plant species, remedy formulations, and dosage. We searched the literature for similarities with other countries and evidence-based pharmacological surveys. The city of Bukavu, capital of South Kivu Province, is located in the eastern part of the Democratic Republic of the Congo (Figure 1), between 2° 3′ of latitude S and 28° 50′ of longitude E at 1600 m of average altitude illustrating a mountainous relief. The climate is tropical humid with a short dry season (3–4 months), and the temperature is moist at the edge of Lake Kivu [15]. Although the city is cosmopolitan, most of the population speaks Swahili, and the dominant ethnolinguistic groups are the Bashi and Lega.

### 2.2. Respondents and Investigation

The respondents' selection started with identifying the traditional practitioners recognized by the Traditional Healers Association operating in the town. We finally selected 18 practitioners working in Bagira, Ibanda, and Kadutu health districts who agreed to deliver their knowledge about using plants treating HT. The information collected focused on the respondent's identity and qualification, the plants' botanical specifications, the preparation of the remedies, and the administration mode. A literature review served to verify whether the plants quoted have similar indications elsewhere and what would be their mechanisms of action.

### 2.3. Statistical Evaluation of the Importance of Species

Three indexes often express the frequency of quoting for botanical families and plant species [16]: the *Relative Frequency of Citation (RFC* = *FC/N; 0* < *RFC* < 1) index, where FC is the number of informants who mentioned the use of the species and N is the total number of informants; *Fidelity Level* [*FL (%)* = *NP/N* × 100] indicating the percentage of informants claiming the use of a specific plant species for the same primary purpose, where Np is the number of informants that claimed use of a plant species to treat a particular disease and N is the number of informants that used plants as a medicine to treat any given disease; and *Use Value (*UV = *ƩU*/*n)*, where *U* is the number of usable reports for a given plant species cited by each informant and *n* is the total number of informants interviewed for a given plant.

## 3. Results

### 3.1. Demographics of Informants

As shown in Table 1, 18 informants participated in the study. They lived in 3 different counties, namely, Bagira (*n* = 7), Kadutu (*n* = 7), and Ibanda (*n* = 4). They qualified themselves as healers (*n* = 4), spiritualists (*n* = 1), and herbalists (*n* = 13). The majority were men (*n* = 14), and a few were women (*n* = 4), aged between 35 and 70 years, practicing for more than ten years.

### 3.2. Ethnopharmacological Information

Table 2 summarizes ethnopharmacological crude data and the calculated importance of each plant. The informants named the plants in their different dialects or scientific names. Qualified botanists of the university helped matching vernacular and scientific names. The analysis of data found 41 plant species cited 165 times belonging to 23 plant families. The fidelity level (FL %) and relative frequency of citation (RFC) and use-value (UV) as defined above varied between 2.5 and 32.5% and 0.056–0.722 and 0.0061–0.0788. A decoction is prepared by boiling 1 to 5 handful(s) of the fresh part or powder in 1 to 2 liters of water for 30 min to 2 hours. Infusions are obtained by soaking the leaves or other parts of the plant in 1-2 liters of boiled water and then filtering the liquid. Based on the types of formulations prepared, the only route of administration is oral. The standard measurement unit is frequently a glass (Gb = 150 ml) generally taken two or three times per day for 5–15 days.

### 3.3. Quantitative Analysis of Data

**Figure 2:**
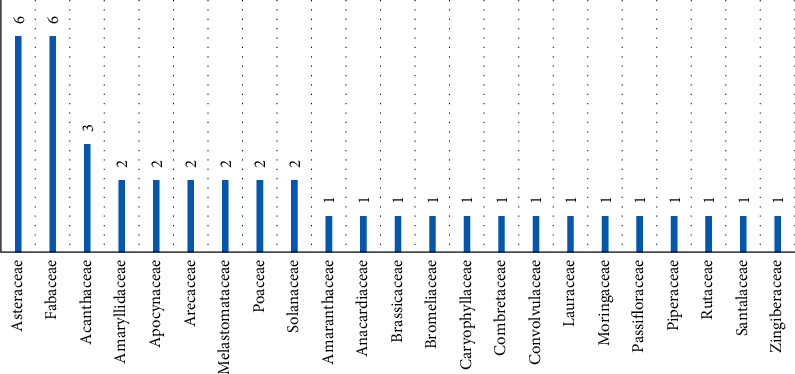
Frequencies of botanical families of antihypertensive plants from Bukavu city.

**Figure 3:**
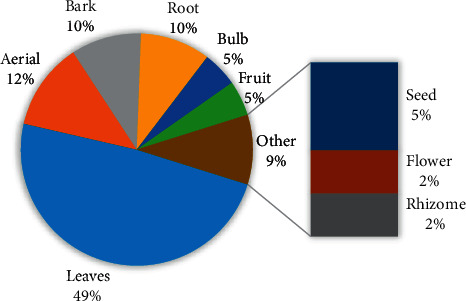
The frequency of plant parts used.

**Figure 4:**
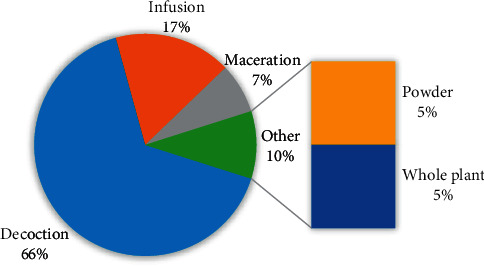
The frequency of remedy preparations.

Figure 2 presents the frequencies of the 23 botanical families. Asteraceae and Fabaceae are the two dominant with six species, each (14.63%). Figures 3 and 4 show the frequency of plant parts used and formulations. The main plant parts used were leaves 20 (48.8%), aerial parts 5 (12.2%), barks 4 (9.8%), and roots 4 (9.8%), formulated as decoction (65.9%), infusion (17.1%), maceration (7.3%) in water, drug powder (4.9%), or crude material (4.9%).

## 4. Literature Discussion

### 4.1. Ethnopharmacological Knowledge

We did not assess the understanding of the interviewees about the physiopathology of hypertension. To our knowledge, based on multiple workshops organized with the association of traditional healers, the hallmark of hypertension is headache, dizziness, and eye redness. For the pharmaceutical state of the art, leaves and aerial parts are the most used parts in formulating remedies. The frequent use of leaves is associated with ease of accessibility among plants' aboveground parts in natural ecosystems [17]. Decoction has often been found as the principal formulation of herbal remedies as it is easy to prepare by mixing a drug with boiling water [18]. The dosing interval and duration of use show that the treatment is more likely for crisis and not for chronic control. Some patients use both conventional and complementary therapies, with an unknown risk of interaction harming.

### 4.2. Ethnobotanical Knowledge

Also, the comprehensive ecological status of individual plants was beyond our objectives. For more details, refer to the references mentioned. The majority of plants listed are of Asteraceae and Fabaceae families. The literature indicates that, out of 250,000 species of flowering plants known, nearly one in ten is a member of the Asteraceae, a diverse family found in almost every habitat in all continents except Antarctica [19]. Bukavu is no exception.

In the consulted literature, the ten top-cited plants were *Allium sativum, Persea americana, Moringa oleifera, Catharanthus roseus, Bidens pilosa, Ageratum conyzoides, Rauvolfia vomitoria, Conyza sumatrensis, Passiflora edulis, Piper capense,* and *Sida rhombifolia.*

### 4.3. The Similarity of Local and Literature Data

Almost all plant species recorded, but 11(26.8%), have validated similar traditional uses in the literature.  Table 3 presents the most frequently cited species. For example, *Galinsoga ciliata, Dissotis trothae, Dyschoriste perrottetii,* and *Hypoestes triflora* were among the highly quoted locally (RFC = 0.556–0.278) but not mentioned as antihypertensive plants in the literature consulted.

*Galinsoga parviflora* Cav, also called gallant soldier, is a cosmopolitan annual herb from the *Asteraceae* family native to South America and a near cosmopolitan weed of distributed places [33]. Fresh leaves and juice of *G. parviflora* have been used in folk medicine worldwide to treat dermatological disorders, including eczema, lichen, and nonhealed bleeding wounds. The use of *G. parviflora* as food by humans for making salad and soups in Latin and North America proves that the plant is nontoxic [34]. *Dissotis trothae* extracts have antidiarrhoeal action [35], and the leaves are used across Africa without strong scientific basis or safety concerns. *Dyschoriste perrottetii* Nees is an important medicinal plant used in various ways to treat microbial infections, fever, measles, and pains [36]. *Hypoestes triflora* aqueous leaf extract showed haematic and hepatoprotective potentials in guinea pigs [37]. *Rauvolfia serpentine is* widely used to manage HT, tachycardia, and thyrotoxicosis since 1952, but respondents did not list it in DRC flora. The ethnomedicine use of *R.vomitoria* is reported only in African countries (Nigeria and Cameroon). For the validated species, there is no more room for debate. Numerous original and review studies discussed multiple uses of *Allium sativum*, *Anacardium occidentalis, Lycopersicum esculentum (Solanum lycopersicum), Persea americana, Ageratum conyzoides,* and *Zingiber officinale* as antihypertensive herbs [17, 20–23, 26, 28, 30–32, 38, 39]. *Moringa oleifera* is well known worldwide [17, 22–24, 26–32, 39–42].

Besides HT treatment, traditional healers use the same plants to manage several diseases [43, 44]. For example, they use *Allium sativum* for abdominal pain, intestinal parasites, infection, and stimulating immunity; *Moringa oleifera* for diabetes, cancer, vomiting, colic, headaches, tooth decay, delirium, inflammation, female infertility, fractures, hemorrhoids, and constipation; *Persea America* for anemia, constipation, kidney, fever, various pains, diarrhea, and sickle cell disease; and *Vernonia amygdalina* for malaria and intestinal worms, to name a few. Also, many plants are often used in association with two to four species. For example, *Erlangea ugandensis* is mixed with piper capense and palm salt.

### 4.4. Evidence-Based Pharmacological Studies

**Figure 5:**
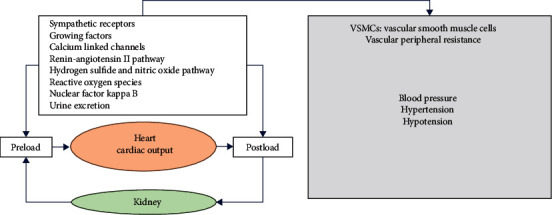
Some modulating mechanisms of blood pressure.

A diverse range of plant and herbal extracts and their metabolites can modulate signaling cascades implicated in the cardiovascular system's physiology (Figure 5). Different authors have explored the pharmacology and toxicology of *Allium sativum* [45–50]. Garlic inhibits ACE activity, and in this regard, gamma-glutamyl-cysteines are the antagonists. The constituents of garlic antagonize vasoconstriction induced by endothelin-1 inhibit the proliferation of VSMCs in smooth muscles and abolish the activation of NF-*κ*B. Numerous studies mention the antihypertensive potentials of *Allium cepa* in animal models through different mechanisms such as increased expression of endothelial nitric oxide synthetase, regulation of extracellular Ca^2+^ levels, attenuation of induced contractions by phenylephrine and KCl, and relaxation of the aorta.

*Bidens pilosa* (Beggar's Tick, Black-Jack, etc.) possesses anticancer, antibacterial, antimalarial, and antiobesity properties alongside the antihypertensive effect [51–54]. Leaf extracts prevented and attenuated HBP in different hypertensive rat models. Cumulative doses of a neutral extract of *B. pilosa* (at an optimum concentration of 0.32 mg/ml) relaxed KCl and noradrenaline preconstricted rat aortas. The mechanism of vasodilation is independent of ATP-sensitive potassium channels; it can involve calcium channel antagonism and cyclooxygenase inhibition.

*Phaseolus lunatus* contains protein hydrolysates with ACE-I Inhibitory Activity [55]. The aqueous extract of ginger (0.05 mg/ml) also inhibited lipid peroxidation and ACE in rat hearts [56]. Besides, zingerone, another active compound in *Zingiber officinale*, can potently scavenge oxidant molecules such as peroxynitrite. In a clinical study, administration of two bioactive constituents of ginger, namely, (6)-gingerol and (6)-shogoal orally (70–140 mg/kg) and intravenously (1.75–3.5 mg/kg), produced triphasic blood pressure profiles: initial rapid fall, intermediate rise, and finally, a delayed decrease in blood pressure [57]. Indeed, (6)-gingerol is now considered a novel angiotensin II type 1 receptor antagonist with an IC50 of 8.17 × 10−6 M [58].

The polyphenol-rich leaf extract of *E. guineensis* has shown vasodilator properties on the aorta and the mesenteric arterial bed such as norepinephrine, mainly via endothelium-dependent mechanisms [59, 60]. It significantly increased serum NO, reduced lipid peroxidation, and showed antioxidant effects in hypertensive rats deficient in NO [59].

*Moringa oleifera* is an analgesic and has anti-inflammatory, antipyretic, anticancer, antioxidant, hepatoprotective, gastroprotective, and antiulcer properties [57, 61–63]. Active constituents for hypotensive action are niazinin A, niazinin B, and niazimicin [63]. The mechanism underlying this cardioprotective activity is the antioxidant effect, the prevention of lipid peroxidation, and the protection of histopathological disturbance [62].

### 4.5. Evidence-Based Toxicological Studies

Overall, toxicological investigations exist mainly in acute and subacute studies in animals, especially rodents. For example, local application of fresh garlic may cause burns (when on the skin, particularly under occlusive dressings). Studies on *Allium* [45, 64] suggested that S-alk(en)yl-l-cysteines have little potential to cause drug-drug interactions through human CYP inhibition or activation. However, garlic may enhance the anticoagulant effect of warfarin and reduce the efficacy of saquinavir in HIV/AIDs patients [64]. Also, the consumption of garlic by nursing mothers may modify their infant's behavior during breastfeeding; it seems contraindicated in pregnant women. Some case reports [64] highlighted garlic allergic reactions such as contact dermatitis, generalized urticaria, angioedema, pemphigus, anaphylaxis, alteration of platelet function, and coagulation with a possible risk of bleeding.

Alkaloids such as 1,2-dehydropyrrolizidine and N-oxides derivatives from *Ageratum conyzoides* could induce hepatotoxicity in humans [65]. Piperine, an amide alkaloid from the genus *Piper*, can depress the central nervous system [66]. *Catharanthus roseus* can be hallucinogenic when taken orally. The known side effects caused by R. serpentine include cardiotoxicity, gastrointestinal disorders, sedation, psychiatric depression, hypotension, nausea, bradycardia, and psychological. Reserpine, a bioactive indole alkaloid, is mainly responsible for these effects [67].

## 5. Conclusions

Traditional healers in Bukavu use many plants validated in the literature in antihypertensive phytotherapy. The plants with high local use-value not backed by other studies deserve in-depth chemical and pharmacological studies to elucidate bioactive compounds and their mechanisms of action.

## 6. Limitations and Perspectives

The list of plants and ethnopharmacological information given here may not be exhaustive due to the small number of informants interviewed. Also, it would be better desirable to contact rural healers who live far from the city. A future specific anthropological survey may help understand the perception of the healers and patients about hypertension, how they feel, symptoms, traditional diagnosis, and what happens when they consume a particular plant. The ultimate end-point of those studies is to come up with improved traditional medicines. Ethically, the authorship claimed by the informants should be regulated organizationally and culturally regarding the international Convention on Biological Diversity.

## Figures and Tables

**Table 1:** Characteristics of informants.

Demographic	District	*N*	%
Address	Bagira	7	38.9
	Kadutu	7	38.9
	Ibanda	4	22.2

Qualification	Healer	4	22.2
	Spiritualist	1	5.6
	Herbalist	13	72.2

Gender	Male	14	77.8
	Female	4	22.2

Age	35–45 years	6	33.3
	46–65 years	9	50.0
	66–70 years	3	16.7

Work experience	10 years	1	5.6
	15 years	3	16.6
	>15 years	14	77.8

**Table 2:** Antihypertensive medicinal plants used by 18 traditional healers in Bukavu.

Scientific name	Botanical family	Local names	FL%	RFC	UV	Parts used	Form preparation		Dose Rx/days
*Acanthus arboreus* Forssk.	Acanthaceae	Ludarhu (Ma)	2.5	0.056	0.0061	Lv	D	2 hf/L	3 × 1 gb/7
*Ageratum conyzoides* (L.) L.	Asteraceae	Munianzigi (Ma)	20.0	0.444	0.0485	AP	D	4 hf/2L	3 × ts/10
*Allium cepa* L.	Amaryllidaceae	Itunguru (Ma)	7.5	0.167	0.0181	Bb	Slice D	Juice 1 hf/2L	Chew as needed
*Allium sativum* L.	Amaryllidaceae	Itunguru sumu (Ma)	32.5	0.722	0.0788	Bb	D	1 hf/2L	2 × 0.5 gb/7
*Anacardium occidentale* L.	Anacardiaceae	Pome (Sw)	2.5	0.056	0.0061	Bk	M	10 g/2L	2 × 1 gb/7
*Ananas comosus* (L.) Merr.	Bromeliaceae	Inanasi (Ma)	2.5	0.056	0.0061	Fr	D	Slide/L	2 × 1 gb/7
*Arachis hypogaea* L.	Fabaceae	Akabemba (Ma) and Kalanga (Sw)	5.0	0.111	0.0121	Lv	D	3 hf/L	2 × 1 gb/7
*Astrochlaena grantii* Rendle	Convolvulaceae	Nfubia (Ma)	2.5	0.056	0.0061	Ro	Pw	Mix + Pw	Lick the Pw
*Bambusa vulgaris* Schrad.	Poaceae	Mulonge (Ma)	10.0	0.222	0.0242	Lv	D	4 hf/2L	1 gb/
*Basella alba* L.	Arecaceae	Nderema (Ma) and Ndelema (Le)	5.0	0.111	0.0121	Lv	I	2 hf/L	2 × 0.5 gb/7
*Bidens pilosa* L.	Asteraceae	Kashisha (Ma)	20.0	0.444	0.0485	AP	D	3 hf/L	3 × 0.5/7
*Brassica oleracea* L.	Brassicaceae	Shu (Sw)	2.5	0.056	0.0061	Lv	I	2 hf/L	As needed
*Capsicum annuum* L.	Solanaceae	Lipipiri (Ma)	5.0	0.111	0.0121	Lv	D	1 kg/2L	2 × 1 gb/
*Cassia alata* L.	Fabaceae		7.5	0.167	0.0181	Bk	I	2 hf/L	2 × 0.5 gb/7
*Cassia didymobotrya* Fresen.	Fabaceae	Mukakabazimu (Ma)	7.5	0.167	0.0181	Lv	D	3 hf/L	2 × 0.5 gb/7
*Catharanthus roseus* (L.) G.Don	Apocynaceae	Vinca (Sw)	15.0	0.333	0.0364	Lv	D	1 hf/L	3 × 1 gb/7
*Chenopodium opulifolium* Schrad. ex W.D.J.Koch & Ziz	Amaranthaceae	Mwigembagembye (Ma)	2.5	0.056	0.0061	Lv	I	2 hf/L	2 × 1 gb/7
*Citrus aurantiifolia* (Christm.) Swingle	Rutaceae		10.0	0.222	0.0242	Lv	I	100 g/2 L	2 × 1 gb
*Conyza sumatrensis* (S.F.Blake) Pruski & G.Sancho	Asteraceae	Mulashahugwe (Ma)	12.5	0.278	0.0303	AP	D	3hf/L	3 × 0.5/7
*Dissotis trothae* Gilg	Melastomataceae	Ciberabana and Ikebya (Ma)	12.5	0.278	0.0303	Bk	M	Pw/L	2 × 1 gb/14
*Drymaria cordata* (L.) Willd. ex Schult.	Caryophyllaceae	Bwahulo (Ma)	10.0	0.222	0.0242	Lv	I	1 ts/L	1 L
*Dyschoriste perrottetii* (Nees) Kuntze	Acanthaceae	Cumumia (Ma)	12.5	0.278	0.0303	Ro	I	2 hf/L	3 × 1 gb/7
*Elaeis guineensis* Jacq.	Arecaceae	Ngazi (Sw)	2.5	0.056	0.0061	Lv	D	3 hf/2 L	1gb/7
*Erlangea ugandensis* S.Moore	Asteraceae	Lwibaye (Ma)	7.5	0.167	0.0181	Ro	Pw	Mix Pw	Lick the Pw
*Galinsoga ciliata* (Raf.) S.F.Blake	Asteraceae	Iragala (Ma)	25.0	0.556	0.0606	AP	D	3 hf/L	2 × 1 gb/7
*Hypoestes triflora* (Forssk.) Roem. & Schult.	Acanthaceae	Mboza (Ma)	12.5	0.278	0.0303	Lv	D	2 hf/L	2 × 0.5gb/7
*Indigofera arrecta* A.Rich.	Fabaceae	Kasholoza (Ma)	7.5	0.167	0.0181	Ro	D	Pw/L	2 × 0.5gb/7
*Kotschya africana* Endl.	Fabaceae	Lwazi (Ma)	7.5	0.167	0.0181	Sm	D	3 hf/3 L	2 × 1gb/7
*Moringa oleifera* Lam.	Moringaceae	Mti maria (Ma) and Mlongelonge (Sw)	25.0	0.556	0.0606	Lv	D	3 hf/2 L	As needed
*Passiflora edulis* Sims	Passifloraceae	Irakucha (Ma)	12.5	0.278	0.0303	Lv	D	5hf/L	3 × 1 gb/
*Persea americana* Mill.	Lauraceae	Mvokati (Ma)	20.0	0.444	0.0484	Lv	D	5 hf/2 L	3 × 1 gb/7
*Phaseolus lunatus* L.	Fabaceae	Kambenga (Ma)	2.5	0.056	0.0061	Sm	D	3 hf/3 L	As needed
*Piper capense* L.f.	Piperaceae	Nkoza (Ma)	17.5	0.389	0.0424	Fr	I	29 gPw/2 L	2 × 1 gb/15
*Rauvolfia vomitoria* Afzel.	Apocynaceae	Katando (Ma)	15.0	0.333	0.0364	Ro	D	Pw/L	As needed
*Sida rhombifolia* L.	Melastomataceae	Mudundu (Ma)	15.0	0.333	0.0364	Lv	Cr	Juice	Chew
*Solanum lycopersicum* L.	Solanaceae	Itomati (Ma)	5.0	0.111	0.0121	Lv	M	200 g/2 L	2 × 1 gb/2
*Terminalia catappa* L.	Combretaceae		2.5	0.056	0.0061	Ro	Pw	Mix Pw	Lick the Pw
*Vernonia amygdalina* Delile	Asteraceae	Mubirizi (Ma)	10.0	0.222	0.0242	AP	D	5 hf/L	3 × 1 gb/7
*Viscum album* L.	Santalaceae		2.5	0.056	0.0061	Lv	D	5 hf/2 L	2 × 1 gb
*Zea mays* L.	Poaceae	Muhindi (Sw)	10.0	0.222	0.0242	Fw	D	1 hf/2 L	2 × 1 ts/
*Zingiber officinale* Roscoe	Zingiberaceae	Tangawizi (Sw)	7.5	0.167	0.0182	Rz	D	2 hfPw/2 L	As needed

Lv, leaves; Rz, rhizome; Fr, fruit; Bk, bark; Ro, root; Fw, flower; Bb, bulb; Sm, stem; AP, aerial part; D, decoction; I, infusion; M, maceration; Pw, powder; Cr, crude; Pc, crushed piece; gb, glass of beer; hf, handful; ts, teaspoon; Ma, Mashi; Le, Lega; Sw, Swahili.

**Table 3:** Frequencies of citation and validation in the literature.

Plant species	Local RFC		Literature^*∗*^RFC		Literature
	Number of respondents	RFC value	Number of papers	RFC value	References
*Galinsoga ciliata*	10	0.556	0	0	Not validated
*Dissotis trothae*	5	0.278	0	0	Not validated
*Dyschoriste perrottetii*	5	0.278	0	0	Not validated
*Hypoestes triflora*	5	0.278	0	0	Not validated
*Allium sativum*	13	0.722	6	0.316	[20–25]
*Persea americana*	8	0.444	5	0.263	[20, 21, 24, 26, 27]
*Moringa oleifera*	10	0.556	4	0.211	[22, 27–29]
*Catharanthus roseus*	6	0.333	4	0.211	[17, 21, 24, 26]
*Bidens pilosa*	8	0.444	2	0.106	[22, 29]
*Ageratum conyzoides*	8	0.444	2	0.106	[18, 21]
*Rauvolfia vomitoria*	6	0.333	2	0.106	[26, 27]
*Conyza sumatrensis*	5	0.278	2	0.106	[23, 30]
*Passiflora edulis*	5	0.278	2	0.106	[23, 31]
*Piper capense*	7	0.389	1	0.052	[26]
*Sida rhombifolia*	6	0.333	1	0.052	[32]
Total sources	18		19		

Relative frequency of citation (RFC).

## Data Availability

The data used in this study are provided and included within the article.
